# The Quality of Infectious Disease Hospital Websites in Poland in Light of the COVID-19 Pandemic

**DOI:** 10.3390/ijerph18020642

**Published:** 2021-01-13

**Authors:** Karol Król, Dariusz Zdonek

**Affiliations:** 1 Department of Land Management and Landscape Architecture, Faculty of Environmental Engineering and Land Surveying, University of Agriculture in Kraków, 253c Balicka Street, 30-149 Kraków, Poland; 2 Department of Economics and Informatics, Faculty of Organization and Management, Silesian University of Technology in Gliwice, 2A AkademickaStreet, 44-100 Gliwice, Poland; dariusz.zdonek@polsl.pl

**Keywords:** website quality, website evaluation, online health information, content quality, covid-19 pandemic, mobile friendliness, web accessibility

## Abstract

The quality of healthcare service websites gains particular importance in the time of the pandemic, asthe popularity of electronic services grows. This applies to infectious disease hospitals as well, often on the front line of the effort against COVID-19. The paper aims to assess the quality of infectious disease hospital websites in Poland in light of the COVID-19 pandemic. The research covered 91 websites. The first stage was an analysis of selected technical attributes of the websites (including website performance, SEO quality, website availability, and mobile-friendliness) with selected online tools, such as Google PageSpeed Insights, Blink Audit Tool, Backlink Checker, andwebsite accessibility evaluation tool (WAVE). The data were then analyzed with statistical methods. The next step was to analyze the content of the websites. The research has shown that most of the websites were of satisfactory quality, apart from those that were not mobile-ready. The following keywords were found most often on the hospital websites: SARS-CoV-2, COVID-19, smear, specialist care clinic, isolation, telephone consultations, sample collection center, support, coronavirus, recommendations, patient registration, signs of disease. The research suggests that the quality of infectious disease hospital websites in Poland is significantly diversified in search engine optimization, mobile-friendliness, and needs of people at risk of digital exclusion.

## 1. Introduction

The number of healthcare providers who appreciate the potential of online services and who have improved their online presence has grown over the recent decade [1]. Their websites are usually intended to provide information, but increasingly often, they offer functionalities for patients and healthcare administration [2,3]. In healthcare, the Internet is used mostly to convey information and improve communication between healthcare providers, but also to increase the availability of medical services and streamline customer service [4]. More and more healthcare institutions transfer some of their services to the Internet to further their often competing goals of increasing the quality of patient care and cost control [5]. Therefore, with the increasing use of the Internet and the dynamic development of e-commerce, customer’srequirements regarding website quality and its evaluation have become a critical success factor for healthcare companies [6].

Most people’s medical expertise is relatively limited. Many find it difficult to comprehend complex intricacies of diseases, details of therapies, or preventive medicine. It is not uncommon for the availability of specialist healthcare services to be limited as well. These factors make informed health decision-making harder. This is why the Internet quickly became one of the most often consulted sources of symptom, treatment, and prevention information [7]. Research shows that adults want to be prepared for medical consultations and can make healthcare decisions based on what they findonline [8]. They look for knowledge on a disease, disorder, or health status online first, and contact a doctor later [9]. A review of websites with medical information can help patients make decisions regarding treatment, a specific doctor, or a healthcare facility [10]. This trend can be expected to accelerate because of the growing prevalence of online mobile devices and number of health-related websites [11]. Some authors believe the impact of medical portals and websites that shape health education, inform about healthcare services, and improve the availability of the services to increase in the future [7,8]. Therefore, improved quality of websites that present and offer medical services may broaden knowledge about healthcare, thus, indirectly, contribute to better health of entire communities [12].

With the COVID-19 pandemic, the quality of information on healthcare and public safety and the quality of their presentation online grew more important. Both public administration websites and websites of healthcare providers play a significant role in this regard. Among them, websites of hospitals with infectious disease wards earmarked for helping patients with the severe acute respiratory syndrome caused by SARS-CoV-2 presenta particular case. Hospital websites should be sources of up-to-date information and should aid the patient-personnel communication process. To this end, they need to conform to numerous quality requirements [13]. Yan et al. [14] looked into how information technology (IT) helped hospitals in mainland China better respond to the outbreak of the pandemic. They analyzed the content of websites of the 50 largest hospitals in continental China from 22 January to 21 February 2020, the early days of the COVID-19 pandemic. They demonstrated that the content could be divided into five major categories: (1) popular medical science education, (2) digitalized hospital processes, (3) knowledge management for medical professionals, (4) telemedicine, and (5) new IT initiatives for healthcare services. The researchers showed that Chinese hospitals published more educational content during the initial stages of the pandemic [14]. The question arises about the quality of websites of infectious disease hospitals in Poland and the content they offer in the times of the COVID-19 pandemic. The purpose of the paper is to assess the quality of infectious disease hospital websites in Poland in light of the COVID-19 pandemic.

This paper is organized as follows. Section 2 describes matters related to the quality of websites. It presents the characteristics of a quality website and lists website attributes that affect its quality. It offers tools and methods for website quality assessment. A review of research on healthcare provider websites follows. These steps facilitate understanding of methods and results in Section 3 and Section 4. Finally, results arediscussed and the paper recapitulated.

## 2. Theoretical Background

The concept of quality is defined by ISO 9000 as the degree to which a set of inherent characteristics of an object fulfils requirements [15]. Website quality is affected by multiple factors and the phenomenon is interdisciplinary in and of itself. It is, therefore, difficult to propose a concise and yet comprehensive definition of website quality as ‘web quality is a vastly undefined concept [16] (p. 468). According to some authors, a uniform notion of website quality is yet to be defined [17].

Website quality is analyzed from various perspectives: that of the user or developer, site usability (user experience) andtechnical attributes (machine experience). Website quality assessment takes into account numerous attributes. Auditors inspect the degree of search engine optimization, content, accessibility, performance, usability, marketing potential, or links but they also use aggregate indices [18].

The quality of a website is considered over many domains. One of the arguments for this proposal is the number of attributes considered when ranking a website on a search engine results page [19,20,21]. According to Rocha [22], website quality can be considered in three domains: content, functionality, and technical aspects. A quality website is loaded quickly [23]. It can be viewed both on desktop computers and mobile devices [24]. The user experience on mobile devices depends on the size and layout of links so that they are spaced sufficiently. For textual links, it is the font color that is important inproviding contrast and readability [25,26]. Content legibility is also one of accessibility criteria, particularly for visually impaired and blind people [27].

A quality website is developed in accordance with the Web Content Accessibility Guidelines (WCAG) [27,28,29]. Website (content) reliability and general usability are important as well [11,30]. As proposed by Devine et al. [11], usability standards can be considered in three main categories: information architecture, site design, and content design. In reality, users believe website design to be among the key indicators of its quality [31].

High-quality websites follow the World Wide Web Consortium (W3C) guidelines regarding code syntax (HyperText Markup Language, HTML and Cascading Style Sheets, CSS) [32] and are loaded quickly [23]. However, the design technique is not all. Quality websites have quality content: it must be valuable, must includemultimedia, and be unique (“content is king”). Texts on a web page should be plain and easy to understand. They should also be appropriately formatted, with headlines, bullet points, and text in bold to improve readability [26]. A website’s address also affects its quality. It should be short and easy to remember [33]. A website of high quality has a clear structure of internal links and a large number of backlinks from quality websites [34]. Moreover, quality is affected by numerous design details. These can includea pre-loader with a system status report, images to facilitate social media use, map or audio components, and small details such as interactive graphics [35].

### 2.1. Tools and Methods for Website Quality Assessment

Recent years have brought an abundance of methods and tools for assessing the quality of websites [18]. Some of them are universal and can be applied to any website. Other ones have been adapted to attributes that are characteristic of specific industries [36]. Website quality assessment methods can be algorithmic, expert-based, or user-based [18]. Algorithmic assessment of website quality most often employs web applications (run in a browser window), browser extensions, such as Lighthouse [37], or software installed on the machine, such as SEO SpyGlass, WebSite Auditor, or Screaming Frog SEO Spider [38]. Emulators, such as Opera Mobile Emulator, are useful tools as well. Website quality is investigated through experiments, with surveys (such as computer-assisted telephone interviews, CATI), and comparative analyses (benchmarking) [6,7].

Website quality assessment employs indicators or indices expressed with numbers, letters, or images. The indices reflect test results in an unambiguous and lucid way [18]. Other methods include checklists and the cognitive walkthrough [39]. Quantitative and qualitative methods are used successfully [5]. Other methods, such as multidimensional scaling and correspondence analysis, weighted scores, simulation, and the index method, have also been used in assessing and improving website quality [5].

The quality of websites can be assessed using models and frameworks as well. One of such tools is WebQual. WebQual covers twelve dimensions: informational fit-to-task; tailored communications; trust; response time; ease of understanding; intuitive operations; visual appeal; innovativeness; emotional appeal; consistent image; online completeness; and relative advantage [40]. Website quality assessment can be performed using the SERVQUAL (ServiceQuality) method proposed by Parasuraman et al. [41] and its variants, HEALTHQUAL for example Lee [42].

### 2.2. Hospital Website Quality and Quality of Medical Content Online

Models and frameworks are often employed to investigate the quality of hospital/medical websites. The criteria of accessibility, content, and apparent features of websites, the design procedure, the graphics applied on the website, and its attractivenesshave been mentioned in the majority of studies [13]. Rafe and Monfaredzadeh [8] proposed a framework for assessing the quality of hospital websites and websites with medical/health content. Their model assesses websites in seven dimensions: content quality, design quality, organization quality, user-friendly quality, performance quality, service quality, and technical points. A paper by Bilsel et al. [5] also presents a model for assessing hospital website performance.

Dulaney et al. [43] investigated the quality of prostate cancer treatment information on cancer center’s websites. They assessed websites of cancer clinics recommended by the National Cancer Institute of the United States of America using a validated decision quality instrument (DQI). They tested the compatibility with mobile devices using the Google mobile-friendly test tool. They demonstrated that websites of cancer centers failed to provide specific information on treatment and its results. The researchers recommended improving the mobile compatibility of the websites and provision of specialist texts in various language versions. They furthermore recommended publication of detailed information about the benefits, results, and toxicity of the presented therapies, also using tables and charts so that multiple therapies could be compared for decision-making. Choi and Lee’s [44] research demonstrated that the usability and functionality of health information portals have increased over the last four years.

Meiyappan et al. [3] investigated information available on websites of pediatric surgery hospitals in Australia and New Zealand. They verified the availability of patient information, including clinical procedural guidelines. They noted the availability of contact details and hospital activity in social media. Research by Meiyappan et al. [3] indicated that the Internet presence of paediatric surgery in Australia and New Zealand was sparse. One-third of the centers did not have hospital web presence. The availability of clinical guidelines and patient information sheets on hospital websites was limited. Mira et al. [45] assessed the quality of websites of Spanish public hospitals, focusing on content readability and accessibility. They investigated 73 attributes using the e-Information Quality Scale of Health Centers. The recommendations included content readability and website availability improvements.

Salarvand et al. [2] evaluated the quality of 59 websites of public hospitals in Tehran. They used a localized checklist, Google page rank, and the Alexa traffic ranking. Their checklist included 112 items in five sections: technical characteristics, hospital information and facilities, medical services, interactive on-line services, and external activities. Data were analyzed using descriptive and analytical statistics. All the websites they investigated were assessed as having unsatisfactory quality (for the research design employed), which confirmed that they needed to be upgraded, and that further studies were necessary.

The purpose of a study by Gümüş and Sönmez [39] was to determine the quality of online services provided by hospitals in Istanbul, Turkey. The first stage involved a survey with 442 residents of Istanbul. The survey focused on preferences for online healthcare services. Then, the authors used the results to build a checklist with four dimensions of attributes: ease of use, credentials of the institution, external activities, and technical features. The findings indicated that, among the top 100 private hospitals in İstanbul, 25% received an excellent rating, while 45% and 35% were ranked as good and fair, respectively. Emmert et al. [46] concluded that online comments failed to reflect the quality of hospital care.

Thus, online ratings are of limited usefulness from a clinical point of view in guiding patients towards high-performing hospitals. Chi et al. [10] assessed the quality and readability of websites with information about sleep apnea and tonsillectomy. They assessed information quality with DISCERN. DISCERN is a brief questionnaire, which offers a valid and reliable way of assessing the quality of written information on treatment choices. Text readability was evaluated with the Flesch-Kincaid Grade Level and Flesch Reading Ease Score. The research demonstrated that a significant portion of published information used excessively expert language and could be hard to understand. Chi et al. [10] concluded that doctors could improve the way they explained therapies with the knowledge on information patients read online [10]. DISCERN was employed also by Killip et al. [47] in their study on the quality, readability, completeness, and accuracy of PTSD (post-traumatic stress disorder) websites for firefighters.

Research often touches upon the readability of websites with medical information [11]. Many researchers have demonstrated that the quality of content on medical websites was below par [48]. They often emphasized that websites lacked information about risks and rewards of therapies and texts were written in expert language, which made them difficult to understand [1,10,49,50]. MacLean et al. [51] believed exhaustive information in plain language to be more readily understandable, which could make it easier for patients to prepare for a procedure, for example. Schreuders et al. [1] investigated the variable quality and readability of patient-oriented websites on colorectal cancer screening. MacLean et al. [51] looked into the readability of online information on colonoscopy preparation. Chi et al. [10] investigated the quality and readability of websites with patient information on tonsillectomy and sleep apnea. Kocyigit et al. [52] investigated the quality and readability of online information on ankylosing spondylitis. The goal of research by Azer et al. [49] was to assess the accuracy of information and readability of websites on kidney and bladder cancer. The readability of the websites was calculated using the Flesch-Kincaid Grade Level Index and the Coleman-Liau Readability Index. Sixty-two websites were finally included in the study. The accuracy and quality of the websites on kidney and bladder cancers varied. In most websites, there were deficiencies in clarity of aims, presenting symptoms, investigations, and treatment options. The majority of them called for improvements.

Llinás et al. [53] assessed the userorientation on hospital websites from Spain, the United States, and the United Kingdom. The study involved 32 websites. They looked into website readability with the Flesch Index, website accessibility with the Web Accessibility Test, and information quality with the e-Information Scale of Health Care Centers. Many websites were found to have accessibility and readability issues [53]. Liu et al. [54] evaluated the quality of websites of 23 hospitals in China. Their research demonstrated that most of the websites were of good quality. The functionality and design quality were satisfactory as well [54]. Perçin [6] investigated the quality of websites of selected Turkish hospitals. In his research, he applied the fuzzy decision-making trial and evaluation laboratory (DEMATEL) method and the generalized Choquet fuzzy integral (GCFI), i.e.,non-additive information fusion technique to aggregate the experts’ preferences and to calculate the overall website quality of hospitals [6]. The purpose of research by Huert et al. [55] was to assess hospital websites in five dimensions: accessibility, content quality, marketing potential, technology, and usability. In their paper, Huert et al. [55] proposed guidelines and design standards to facilitate the use of websites and social media to streamline operations of healthcare providers.

Acosta-Vargas et al. [56] assessed the accessibility of websites of 22 hospitals. Their evaluation was based on WCAG 2.0 guidelines. The research demonstrated the need to improve regulations for implementation of accessibility guidelines on websites of healthcare, public administration, and other institutions funded by the public, in particular.

The objective of the research by Adipridhana et al. [57] was to identify expectations of medical staff of a hospital in Jakarta, Indonesia towards the Portal Hospital Indonesia (PHI) website. They considered such aspects as content quality, navigation structure, design architecture, scope of multimedia available, and unique character of the site. They employed the gap analysis method. It identified discrepancies between personnel expectations and the reality of PHI [57].

The aim of a study conducted by Vetter et al. [58] was to assess the quality of patient information on bariatric surgery available online using the modified Ensuring Quality Information for Patients (EQIP) tool. According to it, the general quality of online information on weight-loss surgery was relatively low. The frequency of complications and related treatment were rarely discussed, even on pages with high EQIP results [58].

The United States Department of Health and Human Services’ Office of Disease Prevention and Health Promotion (ODPHP) conducted a nation-wide evaluation of Web-based health information. Its goal was to determine:(1) a standardized approach to defining and measuring the quality of health websites; (2) benchmarks for measurement; (3) baseline data points to capture the current status of website quality; and (4) targets to drive improvement. The ODPHP devised a tool, the National Quality Health Website Survey, to assess the quality of websites that provide health information [11].

## 3. Materials and Methods

The technical infrastructure of a website can be managed in line with an evolutionary or revolutionary model. The evolutionary model provides regular and consistent modernization of website’s layout, both from the technical point of view (such as updates of scripts, libraries, or code) and graphics (website graphic makeover). In the evolutionary model, upgrades are regular or come in intervals. According to the revolutionary model, a website does not undergo significant upgrades or refreshes. Instead, it is replaced once in a while. A new content management system (CMS) can be implemented every 5 to 7 years, for example. The authors assumed that infectious disease hospital websites are modernized regularly, which is reflected in their usability. The second assumption was that infectious disease hospital websites that are mobile-friendly have the tools to guarantee accessibility, particularly to visually impaired or blind people. The third assumption was that mobile readiness of websites is now a standard feature [24] just as the accessibility of websites of public institutions (funded by the state) [27]. Hence the two research hypotheses:

**Hypothesis** **1**  **(H1).** *values of selected quality attributes of websites depend on the age (date of creation) of the current (valid) version of a website. In other words, new websites offer better quality.*

**Hypothesis** **2**  **(H2).** *a website that is mobile friendly conforms to accessibility criteria.*

Poland is a CentralEuropean country with a population of 38 million people. There are 949 operational hospitals in Poland (according to 2019 data from the Central Statistical Office of Poland). Among them, 150 are private entities [59]. The rest of them are public voivodeship and district (operated by local governments), university, central government, or military hospitals Table 1.

**Figure 1:**
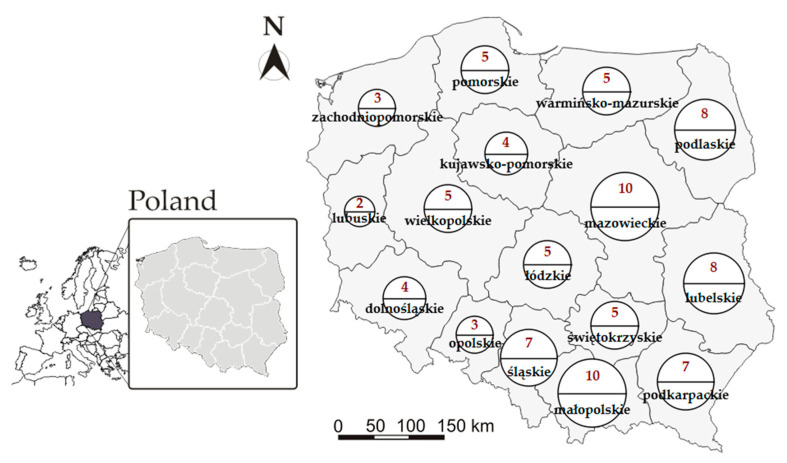
The number of infectious disease hospitals per voivodeship. Source: Original work based on [61].

In 2020, 91 hospitals were transformed into infectious disease hospitals in light of the COVID-19 pandemic. These are private (3) and public (88) specialist units for treating COVID-19 patients. Public centers include military (3), central-government (1), university (7), voivodeship (29), and district (48) hospitals. The hospitals are distributed all over Poland Figure 1.

An infectious disease hospital is a medical facility for treating infectious diseases. Most infectious disease hospitals in Poland have three basic functions: provision of high-quality medical services, prevention of infectious diseases, and medical personnel training. These medical centers have specialist laboratories that offer high-quality modern diagnostics of infectious diseases. Infectious disease hospitals belong to the Polish epidemiological security system.

Information about infectious disease hospitals was retrieved from a register kept by the Ministry of Digital Affairs of the Republic of Poland. The record is published by a government emergency information center for COVID-19 (Coronavirus: information and recommendation) [61]. The register includes COVID-19 laboratories, health and epidemic stations, and nursing homes. Using the register, the authors identified valid website addresses of the hospitals. Only publicly available and free to use data has been analysed and thus exempted from ethical review.

**Figure 2:**
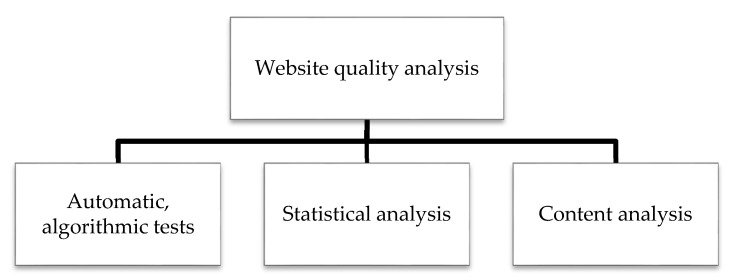
Stages of the research process. Source: original work.

The study on the quality of the 91 infectious disease hospitals’ websites was conducted in three stages Figure 2. The first stage involved investigation of selected technical attributes of the websites using selected online tools. It included accessibility analysis, and verification of mobile compatibility. The data were then analyzed with statistical methods.

The next stage was to verify the content of the websites related to the COVID-19 pandemic. The content was browsed with cognitive walkthrough [63] and COVID-19-related information was noted.

### 3.1. Website Quality Analysis

The hospital websites were analyzed using four criteria: load time, search engine optimization (SEO), accessibility, and mobile friendliness. The analysis employed selected online tools:

- website performance was measured with Google PageSpeed Insights (PSI) [64]
- the value of the SEO attribute was measured with the Blink Audit Tool [65]. The analysis was expanded with a verification of the number of bad backlinks with Backlink Checker [66]
- website availability was verified with the WAVE Web Accessibility Evaluation Tool [67]
- website mobile friendliness was verified with the Mobile-Friendly Test (Bulk Testing Tool) application [68].

Table 2 offers a complete list of all the tested attributes. The attributes for the evaluation of the website quality were selected based on results of past research [2,3,18,37,43,45,54,55,56]. The list was then expanded with new aspects such as SEO audit, link audit, or the age of the websites. Moreover, the authors investigated content of the websites for COVID-19 information.

The authors paid attention to other website components that can affect websitequality, such as HTML specification and the CMS. Therefore, the analysis involved not only content displayed in the viewport but also the source code of each page Table 3.

The cognitive walkthrough verified two quality attributes related to website usability and accessibility for visually impaired persons (WCAG): font size adjustment and contrast adjustment. The authors further analyzed the development path of the website over the last 5 years using archived copies in the Internet Archive [69]. This step facilitated determination of the year the current website version was created.

The results are presented in figures and tables. Results were presented graphically as a word cloud. Keywords in a set are usually displayed using a larger font. The word cloud was generated using the Word Cloud Generator web application [70].

### 3.2. Statistical Analysis

The complete data were analyzed statistically with Microsoft Excel 2013 (Microsoft, Warsaw, Poland) and STATISTICA 13.3 (StatSoft, Krakow, Poland). The authors performed a statistical analysis to calculate descriptive statistics of selected variables and investigated correlation between data produced by selected diagnostic tools.

The descriptive statistical analysis focused mostly on calculating such quantitative measures as the arithmetic mean, standard deviation and median, coefficient of variation, and minimum and maximum values. Statistical hypotheses were verified using statistical tests, taking into account significance at the level of α≤0.05. At the same time, it was noted that the p probability of a type I error was not greater than 0.05. In the correlation analysis, the linear Pearson correlation coefficient was used.

## 4. Results

The study investigated the quality of websites of infectious disease hospitals (n = 91). Thirty-four websites (about 38%) presented information about their authors (usually a logo with a link). The analysis of the source code of the websites identified several various CMSs, including WordPress (35 websites, 39%), Joomla (16 websites, 18%), TYPO3 (2 websites), PHP-Fusion (1 website), and Drupal (1 website). Thirty-six websites (about 38%) used other CMSs or content management technologies that could not be identified through source code exploration.

Most of the websites followed the HTML5 specification (80%), which corresponds with the number of websites using CMSs. The XHTML specification was used on 16 websites (about 18%). The other websites were designed in HTML 4.01 (about 2%). Half of the websites used secure HyperText Transfer Protocol (HTTPS, data encryption using the SSL/TLS protocol). It may mean that merely half of the websites offered advanced functionalities that require encrypted communication such as sending data through online forms.

The hospital websites were created fairly recently and were 4.67 ± 2.6 years old. Website age was noted as integers based on analysis of data in the Internet Archive. A website built in 2020 scored 1 point, which meant it has been functional for a year. The maximum score was 11 points, which means the hospital has been using the website since 2009.

Most hospital’s websites were up to five years old. Note that 17 hospitals replaced their websites in 2018 Figure 3.

Most of the websites were mobile friendly. Sixty-six websites (about 73%) passed the Bulk Testing Tool mobility test. At the same time, the websites offered relatively poor browsing comfort for mobile devices according to algorithmic tests Table 4. The most common issues were: “use legible font size” (78% of the websites) and “tap targets too close” (about 73% of the websites).

**Figure 4:**
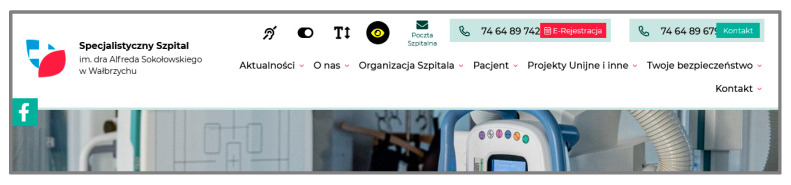
A piece of a website with tools for changing font size and contrast. Source: website of Alfred Sokołowski Specialist Hospital in Wałbrzych.

Most of the websites had to be optimized in terms of content accessibility. The tools identified 17.32 ± 19.6 accessibility errors on average (min. = 0, max. = 142). Moreover, the average number of accessibility contrast errors was 23.75 ± 19.6 (min. = 0, max. = 244) with 49.03 ± 61 (min. = 2, max. = 310) accessibility alerts. The cognitive walkthrough helped find functions for enlarging the font and adjusting contrast. In most cases, they were buttons, i.e., images in the upper part of the website Figure 4. Contrast adjustment was possible for 51 websites (about 56% of websites), while font size could be changed on 55 websites (about 60%).

The performance of the websites was tested with Page Speed Insights in the desktop and mobile modes. The attribute assumed values between 0 and 100. The mean mobile performance of the websites was 40.87 ± 25.1 units (min. = 1, max. = 100). The desktop performance was 63.65 ± 21.4 units (min. = 7, max. = 100). Note that according to a Google recommendation, 0–49 PSI performance is “poor.” Performance of 50–89 is “satisfactory”, but any such website is annotated “needs improvement.” Most of the websites scored relatively low on the PSI test, particularly on mobile devices Table 5.

The search engine optimization (SEO) analysis employed the Blink Audit Tool. The application provides SEO audit results in two dimensions, general SEO evaluation and SEO for marketing (so-called semantic result). The mean general SEO score for the investigated websites was 60.69 ± 9.00 (min. = 43, max. = 86), while the mean semantic score was 63.93 ± 12.40 (min. = 40, max. = 90). Although this result is fairly satisfactory, it shows the significant untapped SEO potential of the websites and the need for optimization of many of them.

The SEO analysis was followed by verification of the number of broken backlinks according to the Backlink Checker Tool. The number of bad links varied significantly from 0 to 3,430,561. The authors then analyzed user opinions for each hospital and the numbers of opinions. The average score was 2.84 ± 0.6 (min. = 1.6, max. = 5). The mean number of opinions was 111.14 ± 97.9 (min. = 5, max. = 518).

### 4.1. Correlation Analysis

A statistical analysis with Statistica demonstrated significant correlations for ten pairs of variables Table 6. A strong correlation was noted between Mobile PageSpeed Insights (G_mps) and Desktop PageSpeed Insights (G_dps). It means that the performance of the websites measured with the PSI tool was similar for desktop and mobile devices. Another strong correlation was identified for the website age according to the Internet Archive (attribute Year) and its performance (G_mps, G_dps). This means that latest versions of the hospital websites created over recent years loaded slower in browser window, which resulted in lower PSI values.

A weak but significant correlation was noted between the website age (attribute Year) and the number of contrast errors according to WAVE (attribute W_aim_c). It may mean that more recent websites have more functionalities to accommodate people with impaired vision. No correlation was identified between hospital scores on Google and attributes of their websites. User opinions on Google concerned mostly the quality of medical services provided, patient care, and also cleanliness, registration time, communication availability, and so on. No Google opinion related to a hospital website was found.

### 4.2. Content Analysis

**Figure 5:**
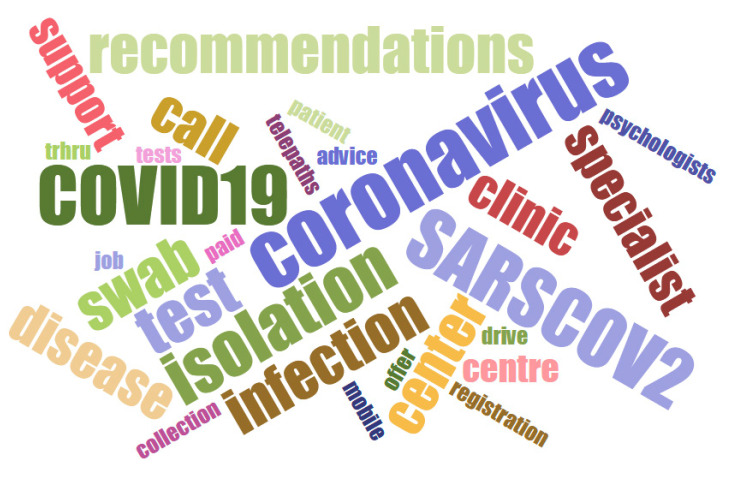
Word cloud–the frequency of selected keywords related to the COVID-19 pandemic on websites of infectious disease hospitals in Poland.

The primary topic on the hospital websites was the coronavirus and matters related to COVID-19. This factwas clear from the images used on the websites and keywords in texts Figure 5. The most common keywords included: SARS-CoV-2, COVID-19, smear, specialist care clinic, isolation, telephone consultations, sample collection facility, paid testing, support, gratitude, mobile testing centers, coronavirus, recommendations, patient registration, signs of disease.

Buttons linking to pages with COVID-19 information could be found on 33 websites. The pages were FAQs related to the pandemic. The most common words used to describe the image leading to the FAQs were coronavirus and COVID-19. Twenty-nine websites (about 32%) had a tab dedicated to functional changes in the hospitals due to the pandemic. In most cases (64 websites, about 70%) information on changed work organization in the hospitals was posted in the news section.

The COVID-19 pandemic caused many organizational changes in hospitals. The hospitals informed the public via their websites most often about the following:

- limited or cancelled consultations in specialist clinics (59 hospitals, about 65% of the websites)
- increased demand for medical and technical personnel; the home page had job advertisements for volunteers, nurses, paramedics, doctors, and technical staff (42 hospitals, about 46%)
- suspension of hospital visiting (35 hospitals, about 39% of the websites). Moreover, many hospitals informed the public about restricted movement within hospital complexes, restricted visiting (if possible at all), and limits of people allowed in waiting rooms and corridors
- suspension of specific medical procedures (14 hospitals, about 15% of the websites)
- need for convalescent plasma (9 hospitals), donated by recovered patients
- in eight cases, hospitals informed about delayed, limited, or suspended access to medical documentation.

Moreover, care for COVID-19 patients caused several organizational changes in hospitals. Specialist clinics work organization changed. Some hospitals suspended residency training programs and training for students. Some hospitals extended personnel work hours.

Relatively few hospitals, 12, opened special hotlines (telephone consultations, mental counselling) and informed the public about theseon their websites. Thirty-four pages (about 37%) contained thank-you notes for donors or sponsors and medical personnel. Fourteen websites (about 15%) offered commercial, paid SARS-CoV-2 tests. Information about mobile SARS-CoV-2 testing facilities was found on 23 hospital websites. No fewer than 33 hospitals (about 36%) asked for financial donations on their websites.

## 5. Discussion

Jeddi et al. [13] found such aspects as website accessibility, performance, content readability, graphical appeal, and security of personal data to be critical criteria for assessing website quality regardless of the owner. Industry-specific websites may have unique characteristics different than other portals. Therefore, website quality assessment should consider the nature of the industry, website type, and its target.

The Internet is a popular source of information about symptoms, treatments, or opinions about doctors for people suffering from medical conditions. They often search for others with similar diseases to form support groups and exchange experiences. These phenomena have grown over the recent decade as mobile devices became more readily available. Research shows that a significant portion of online health information is presented in an unreliable way. Additionally, many Internet users lack the knowledge and skills necessary to tell between reliable and fake information [71].

Yan et al. [14] have demonstrated that hospitals in China published mainly educational content to prevent the spread of SARS-CoV-2 at the early stage of the pandemic. In Poland, websites of infectious disease hospitals proposed more advisory content in November 2020 for those who had contact with an infected person, those were looking to be tested for coronavirus, or people with symptoms. This shows that the content of information on hospital websites changed with time and as the pandemic spread [14].

Moreover, access to the information can be limited by mobile incompatibility or excessively sophisticated, expert language. According to Cheng and Dunn [72], mobile friendliness should be a priority for organizations that publish health information online. The same observationpertains to health and lifestyle portals, websites of medical service providers, and websites of healthcare facilities.

There is no single, universal method for assessing the quality of hospital websites. The algorithmic quality assessment takes into account many attributes, including technical and visual ones. The evaluated attributes change over time, as the World Wide Web evolves, and have various impacts on the final score [73]. Hence the importance of ongoing testing and search for quality assessment criteria as well as new diagnostic methods and tools. The literature research and empirical studies have demonstrated that the performance, search engine optimization (SEO), accessibility (WCAG), and mobile friendliness are important factors in website quality assessment.

Most of the websites were of satisfactory quality apart from those that were not mobile-friendly. Such websites can be browsed virtually only on desktop or laptop computers. It is, of course, possible to display such a website in a mobile browser, but the user experience is extremely poor or it is impossible to browse them at all.

Polish public institution websites need to be accessible by law [27]. This is why hospital websites have such tools as contrast or font size adjustment. Regrettably, website accessibility is approached mechanically in many cases just to satisfy certain minimum regulatory requirements.

Detailed information displayed on a quality website raises confidence [71]. Reliable and exhaustive information available through online channels can reduce the bulk of e-mail and telephone questions to the medical staff regarding organizational matters. It is, therefore, in the best interest of hospitals to publish information about current changes, organizational ones in particular, as thismay relieve personnel busy with ongoing patient service on site (registration, reporting, statistics, and administration in general).

All the investigated websites had published announcements related to the COVID-19 pandemic. Significant differences were evident in the frequency of publication and the frequency of announcement updates. Thismay mean that some hospitals have managers and marketing and PR departments that take care of the hospital’s image. Content management can also be the job of technical personnel, particularly the IT department.

The study has shown that the first hypothesis was true (H1); values of selected quality attributes of websites are correlated with the hospital website age. It is particularly true for the performance of new websites, which may need optimization. The optimization most often involves code minification and compression of images and content management system extensions (for example, fancy widgets). The study has proven the second research hypothesis (H2) false. No significant relation was identified between mobile friendliness and accessibility (under the employed research design).

## 6. Conclusions

The quality of infectious disease hospital websites in Poland varies significantly, in terms of search engine optimization, mobile friendliness, and needs of people at risk of digital exclusion. The websites have different technical frameworks and content management approaches. Among the investigated websites, some were updated relatively rarely, but others had several to over a dozen announcements a month. This leads to a conclusion that website quality derives from technical aspects, but also the actions of the content manager. Personnel shortages and lack of a content editor lead to a poorer use of the potential of electronic communication channels.

Websites are important channels of communication between hospitals and their socioeconomic environment. Gradually changing the ownership structure of hospitals and private competitors drives more advanced forms of online promotion. Today, the vast majority of Polish hospitals arepublicly funded, and the demand for healthcare services exceeds the supply; hence, not all hospitals tend to their online image. The quality of hospital websites can be improved to some extent with regulations such as guidelines for accessibility and privacy policy. Adaptation of websites to conform to the requirements often results in a complete layout change and modification of content management methods. Many hospital websites employ content management systems because thesearesomeof the most effective ways of changing a website and conforming to the latest standards. At the same time, such matters as website performance optimization and search engine optimization tend to be neglected.

### Practical Implications and Limitations of the Research

The research has indirectly demonstrated the importance of technical and administration personnel responsible for electronic communication with patients. The high quality of a website is not set in stone. Content needs to be updated in line with the dynamics of events and changing circumstances. Still, it is people who publish content. This shows that apart from medical personnel, the technical staff, IT, and administration are important components of the hospital ecosystem. This personnel is responsible for the quality of medical services, the image of the facility, and public communication.

The research evaluated selected website quality attributes. A larger pool of assessed attributes would facilitate the better evaluation of the quality of the websites. On the other hand, the authors conducted a SEO audit with a tool, which aggregates results of multi-dimensional tests (partial tests). One can always increase the number of assessed quality attributes to obtain more precise results. The research focused on analyzing the content of home pages, especially news sections. More precise results can also be obtained by extracting texts from all pages of a website. This approach would require a crawler for fetching the data and an algorithm that could analyze it. This is a good starting point for future research. Moreover, research with computer tools can be complemented with a survey among Internet users.

## Figures and Tables

**Figure 3:**
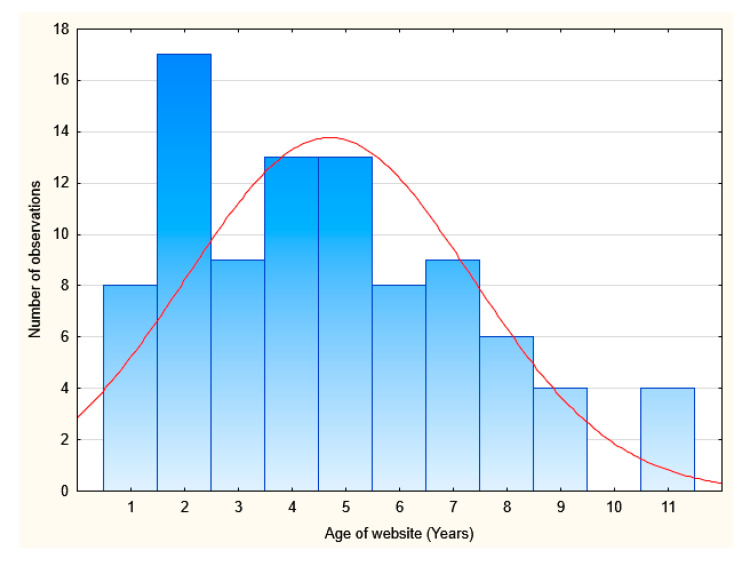
The age of hospital’s websites. Source: Original work based on the Internet Archive.

**Table 1:** The number of hospitals and hospital beds per voivodeship.

Voivodeship	No. of Infectious Disease Hospitals	Total Hospitals	Hospital Beds (Thousand)	Residents (Thousand)
Dolnośląskie	4	82	15	2900
Kujawsko-pomorskie	4	41	10	2072
Lubelskie	8	45	11	2108
Lubuskie	2	24	4	1012
Łódzkie	5	69	13	2455
Małopolskie	10	88	15	3411
Mazowieckie	10	121	26	5423
Opolskie	3	30	4	983
Podkarpackie	7	42	10	2127
Podlaskie	8	34	6	1178
Pomorskie	5	45	9	2344
Śląskie	7	157	25	4518
Świętokrzyskie	5	24	6	1234
Warmińsko-mazurskie	5	43	6	1423
Wielkopolskie	5	61	15	3499
Zachodniopomorskie	3	43	8	1696
Total	91	949	183	38,383

Source: original work based on [60,61,62]. As on 20.11.2020.

**Table 2:** List of attributes and their symbols.

Id	Hospital Identifier
woj	Voivodeship
www	Hospital website URL
G_onr	Google–number of opinions
G_oav	Google–mean opinion value
G_mps	Google–Mobile PageSpeed Insights value
G_dps	Google–Desktop PageSpeed Insights value
S_seo	Blink Audit Tool–SEO value
S_seos	Blink Audit Tool–semantic SEO value
W_aim_e	WAVE accessibility–Errors
W_aim_c	WAVE–contrast Errors
W_aim_a	WAVE–Alerts
B_blk	Backlink Checker–number of broken backlinks
Mr_mf	Mobile readiness–Mobile-Friendly
Mr_cv	Mobile readiness–Configure Viewport
Mr_fvw	Mobile readiness–Fixed Width Viewport
Mr_sctv	Mobile readiness–Size Content To Viewport
Mr_ulfs	Mobile readiness–Use Legible Font Sizes
Mr_tttc	Mobile readiness–Tap Targets Too Close
Mr_uip	Mobile readiness–Uses Incompatible Plugins
Ec	External company
CMS	CMS designation
HTML	HTML standard
W_contr	Website contrast adjustment (accessibility feature)
W_font	Website font adjustment (accessibility feature)
S_http	Encrypted communication (http://, https://)
Years	The number of years the new website has been available (analyzed in the Internet Archive)
Comments	Comments for websites made during the cognitive walkthrough

Source: original work.

**Table 3:** A piece of source code of a website.

<head>(…)<link rel = ‘stylesheet’ id = ‘wp-block-library-css’ href = ‘http://zeromski-szpital.pl/**wp-includes**/css/dist/block-library/style.min.css?ver = 5.5.1’ type = ‘text/css’ media = ‘all’ /> <link rel = ‘stylesheet’ id = ‘contact-form-7-css’ href = ‘http://zeromski-szpital.pl/wp-content */plugins/contact-form-7/includes/css/styles.css?ver = 5.2.2’ type = ‘text/css’ media = ‘all’ />(…) </head>

* The bolded piece of the URL ‘wp-content’ means that the website was created using WordPress.Source: a piece of source code of the website of Stefan Żeromski Specialist Hospital, Independent Public Healthcare Facility in Kraków.

**Table 4:** “Mobile friendly” test results.

Designation	Description	Number of Websites	Percentage (%)
Mr_cv	Configure Viewport	78	86
Mr_fvw	Fixed Width Viewport	91	100
Mr_sctv	Size Content to Viewport	86	95
Mr_ulfs	Use Legible Font Sizes	71	78
Mr_tttc	Tap Targets too Close	66	73
Mr_uip	Uses Incompatible Plugins	91	100

**Table 5:** Results of Google PageSpeed Insights (PSI) performance tests.

Test Range	Good (>89)	Needs Improvement (50–89)	Poor (0–49)
PSI mobile–G_mps (%)	3 (3%)	30 (33%)	58 (64%)
PSI desktop–G_dps (%)	13 (14%)	55 (60%)	23 (25%)

**Table 6:** Correlations between website quality assessment attributes.

Id.	AVE	SD	G_oav	G_mps	G_dps	S_seo	S_seos	W_aim_e	W_aim_c	W_aim_a	Year
G_oav	2.84	0.6	1.00								
G_mps	40.87	25.1	−0.10	1.00							
G_dps	63.65	21.4	−0.11	0.89	1.00						
S_seo	60.69	9.0	−0.09	0.08	0.20	1.00					
S_seos	63.93	12.4	0.02	0.10	0.12	0.55	1.00				
W_aim_e	17.32	19.6	0.19	−0.22	−0.28	−0.28	−0.07	1.00			
W_aim_c	23.75	41.6	0.18	−0.22	−0.25	0.09	0.13	0.14	1.00		
W_aim_a	49.03	61.0	0.08	0.09	0.12	0.14	0.14	0.10	0.12	1.00	
Year	4.67	2.6	0.01	0.66	0.55	0.04	0.06	−0.02	−0.28	−0.04	1.00

G_oav–the mean Google opinion value; G_mps–Mobile PageSpeed Insights; G_dps–Desktop PageSpeed Insights; S_seo–the general search engine optimization (SEO) score by the Blink Audit Tool; S_seos–the semantic SEO score by the Blink Audit Tool; W_aim_e–web accessibility evaluation tool (WAVE)–Errors; W_aim_c–WAVE–contrast Errors; W_aim_a–WAVE–Alerts; Year–the age of the current version of the website according to the Internet Archive; Note: *p* < 0.05 (n = 91), linear Pearson correlation coefficients.

## Data Availability

The datasets generated and analyzed during the current study are not publicly available due to participant confidentiality but are available from the corresponding author on reasonable request.

## References

[1] Schreuders E.H., Grobbee E.J., Kuipers E.J., Spaander M.C.W., van Zanten S.J.O.V. (2017). Variable Quality and Readability of Patient-Oriented Websites on Colorectal Cancer Screening. Clin. Gastroenterol. Hepatol..

[2] Salarvand S., Samadbeik M., Tarrahi M.J., Salarvand H. (2016). Quality of public hospitals websites: A cross-sectional analytical study in Iran. Acta Inf. Med..

[3] Meiyappan V., Little T.A., Jackson P. (2019). Evaluation of Website Information Provided by Paediatric Surgery Centres in Australia and New Zealand. ANZ J. Surg..

[4] Cebi S. (2013). Determining Importance Degrees of Website Design Parameters Based on Interactions and Types of Websites. Decis. Support. Syst..

[5] Bilsel R.U., Büyüközkan G., Ruan D. (2006). A Fuzzy Preference-Ranking Model for a Quality Evaluation of Hospital Web Sites. Int. J. Intell. Syst..

[6] Perçin S. (2019). A Combined Fuzzy Multicriteria Decision-Making Approach for Evaluating Hospital Website Quality. J. Multi-Criteria Decis. Anal..

[7] Bujnowska-Fedak M.M., Węgierek P. (2020). The Impact of Online Health Information on Patient Health Behaviours and Making Decisions Concerning Health. Int. J. Environ. Res. Public Health.

[8] Rafe V., Monfaredzadeh M. (2012). A Qualitative Framework to Assess Hospital/Medical Websites. J. Med. Syst.

[9] Hesse B.W., Nelson D.E., Kreps G.L., Croyle R.T., Arora N.K., Rimer B.K., Viswanath K. (2005). Trust and sources of health information: The impact of the Internet and its implications for health care providers: Findings from the first Health Information National Trends Survey. Arch. Intern. Med..

[10] Chi E., Jabbour N., Aaronson N.L. (2017). Quality and Readability of Websites for Patient Information on Tonsillectomy and Sleep Apnea. Int. J. Pediatr. Otorhinolaryngol..

[11] Devine T., Broderick J., Harris L.M., Wu H., Hilfiker S.W. (2016). Making Quality Health Websites a National Public Health Priority: Toward Quality Standards. J. Med. Internet Res..

[12] Fiksdal A.S., Kumbamu A., Jadhav A.S., Cocos C., Nelsen L.A., Pathak J., McCormick J.B. (2014). Evaluating the process of online health information searching: A qualitative approach to exploring consumer perspectives. J. Med. Internet Res..

[13] Jeddi F.R., Gilasi H., Khademi S. (2017). Evaluation models and criteria of the quality of hospital websites: A systematic review study. Electron. Physician.

[14] Yan A., Zou Y., Mirchandani D.A. (2020). How Hospitals in Mainland China Responded to the Outbreak of COVID-19 Using Information Technology–Enabled Services: An Analysis of Hospital News Webpages. J. Am. Med. Inf. Assoc..

[15] ISO9000: 2015 Quality Management Systems—Fundamentals and Vocabulary. Polish Committee for Standardization. https://sklep.pkn.pl/pn-en-iso-9000-2015-10p.html.

[16] Aladwani A.M., Palvia P.C. (2002). Developing and Validating an Instrument for Measuring User-Perceived Web Quality. Inf. Manag..

[17] Semerádová T., Weinlich P., Semerádová T., Weinlich P. (2020). Looking for the Definition of Website Quality. Website Quality and Shopping Behavior: Quantitative and Qualitative Evidence.

[18] Król K., Zdonek D. (2020). Aggregated Indices in Website Quality Assessment. Future Internet.

[19] Schubert D. (2016). Influence of mobile-friendly design to search results on Google search. Procedia-Soc. Behav. Sci..

[20] Rieder B., Matamoros-Fernández A., Coromina Ò. (2018). From Ranking Algorithms to ‘Ranking Cultures’: Investigating the Modulation of Visibility in YouTube Search Results. Convergence.

[21] Gao R., Shah C. (2020). Toward creating a fairer ranking in search engine results. Inf. Process. Manag..

[22] Rocha Á. (2012). Framework for a global quality evaluation of a website. Online Inf. Rev..

[23] Gajanova L., Krizanova A., Lăzăroiu G., Tsounis N., Vlachvei A. (2020). Analysis of Website Performance Dependence on Global Brand Value. Advances in Cross-Section Data Methods in Applied Economic Research.

[24] Błażejczyk I., Trawiński B., Indyka-Piasecka A., Kopel M., Kukla E., Bernacki J., Nguyen I., Manolopoulos L., Trawiński Y. (2016). Usability Testing of a Mobile Friendly Web Conference Service. Computational Collective Intelligence.

[25] Kous K., Polančič G. (2019). Empirical Insights of Individual Website Adjustments for People with Dyslexia. Sensors.

[26] Ashraf N., Faisal M.N., Jabbar S., Habib M.A. (2019). The Role of Website Design Artifacts on Consumer Attitude and Behavioral Intentions in Online Shopping. Tech. J..

[27] Król K., Zdonek D. (2020). Local Government Website Accessibility–Evidence from Poland. Adm. Sci..

[28] Fernández-Díaz E., Iglesias-Sánchez P.P., Jambrino-Maldonado C. (2020). Exploring WHO Communication during the COVID 19 Pandemic through the WHO Website Based on W3C Guidelines: Accessible for All?. Int. J. Environ. Res. Public Health.

[29] Acosta-Vargas P., Hidalgo P., Acosta-Vargas G., Gonzalez M., Guaña-Moya J., Salvador-Acosta B., Ahram T., Karwowski W., Vergnano A., Leali F., Taiar R. (2020). Challenges and Improvements in Website Accessibility for Health Services. Intelligent Human Systems Integration 2020.

[30] Ratwani R.M., Reider J., Singh H. (2019). A Decade of Health Information Technology Usability Challenges and the Path Forward. JAMA.

[31] Weymann N., Harter M., Dirmaier J. (2015). Quality of Online Information on Type 2 Diabetes: A Cross-Sectional Study. Health Promot. Int..

[32] West A.W., West A.W. (2016). Go Live and Validate Your Website. Practical Web Design for Absolute Beginners.

[33] Moro Visconti R., Moro Visconti R. (2020). Domain Name and Website Valuation. The Valuation of Digital Intangibles: Technology, Marketing and Internet.

[34] Król K., Zdonek D. (2019). Peculiarity of the bit rot and link rot phenomena. Glob. Knowl. Mem. Commun..

[35] Wang B., Jin D., Lin S. (2012). The Research of Web2.0 Interface and Interaction. Advances in Electronic Engineering, Communication and Management.

[36] Morales-Vargas A., Pedraza-Jiménez R., Codina L. (2020). Website Quality: An Analysis of Scientific Production. Prof. De La Inf..

[37] Giannakoulopoulos A., Konstantinou N., Koutsompolis D., Pergantis M., Varlamis I. (2019). Academic Excellence, Website Quality, SEO Performance: Is There a Correlation?. Future Internet.

[38] Shenoy A., Prabhu A., Shenoy A., Prabhu A. (2016). SEO Hub: Utilities and Toolsets. Introducing SEO: Your Quick-Start Guide to Effective SEO Practices.

[39] Gümüş R., Sönmez Y. (2020). Quality of Online Communication Tools at Hospitals and Their Effects on Health Service Consumers’ Preferences. Int. J. Healthc. Manag..

[40] Loiacono E.T., Watson R.T., Goodhue D.L. (2007). WebQual: An Instrument for Consumer Evaluation of Web Sites. Int. J. Electron. Commer..

[41] Parasuraman A., Zeithaml V.A., Berry L.L. (1988). SERVQUAL: A multi-item scale for measuring consumer perceptions of service quality. J. Retail..

[42] Lee D. (2017). HEALTHQUAL: A multi-item scale for assessing healthcare service quality. Serv. Bus..

[43] Dulaney C., Barrett O.C., Rais-Bahrami S., Wakefield D., Fiveash J., Dobelbower M. (2016). Quality of Prostate Cancer Treatment Information on Cancer Center Websites. Cureus.

[44] Choi H., Lee S.-K. (2020). A Prospective Analysis of Health Information Portals in Four Years. Int. J. Environ. Res. Public Health.

[45] Mira J.J., Llinás G., Tomás O., Pérez-Jover V. (2006). Quality of Websites in Spanish Public Hospitals. Med. Inf. Internet Med..

[46] Emmert M., Meszmer N., Schlesinger M. (2018). A Cross-Sectional Study Assessing the Association between Online Ratings and Clinical Quality of Care Measures for US Hospitals: Results from an Observational Study. BMC Health Serv. Res..

[47] Killip S.C., Kwong N.K.R., MacDermid J.C., Fletcher A.J., Carleton N.R. (2020). The Quality, Readability, Completeness, and Accuracy of PTSD Websites for Firefighters. Int. J. Environ. Res. Public Health.

[48] Cheneguin A.A., Salvat I.S., Barrero H.R., Lacomba M.T. (2020). How Good Is Online Information on Fibromyalgia? An Analysis of Quality and Readability of Websites on Fibromyalgia in Spanish. BMJ Open.

[49] Azer S.A., Alghofaili M.M., Alsultan R.M., Alrumaih N.S. (2018). Accuracy and Readability of Websites on Kidney and Bladder Cancers. J. Cancer Educ..

[50] Arts H., Lemetyinen H., Edge D. (2020). Readability and Quality of Online Eating Disorder Information—Are They Sufficient? A Systematic Review Evaluating Websites on Anorexia Nervosa Using DISCERN and Flesch Readability. Int. J. Eat. Disord..

[51] MacLean S.A., Basch C.H., Clark A., Basch C.E. (2018). Readability of Information on Colonoscopy Preparation on the Internet. Health Promot. Perspect..

[52] Kocyigit B.F., Koca T.T., Akaltun M.S. (2019). Quality and readability of online information on ankylosing spondylitis. Clin. Rheumatol..

[53] Llinás G., Rodríguez-Iñesta D., Mira J.J., Lorenzo S., Aibar C. (2008). A Comparison of Websites from Spanish, American and British Hospitals. Methods Inf. Med..

[54] Liu X., Bao Z., Liu H., Wang Z. (2011). The Quality and Characteristics of Leading General Hospitals’ Websites in China. J. Med. Syst..

[55] Huerta T.R., Hefner J.L., Ford E.W., McAlearney A.S., Menachemi N. (2014). Hospital Website Rankings in the United States: Expanding Benchmarks and Standards for Effective Consumer Engagement. J. Med. Internet Res..

[56] Acosta-Vargas P., Acosta T., Luján-Mora S. Framework for Accessibility Evaluation of Hospital Websites. Proceedings of the 2018 International Conference on eDemocracy eGovernment (ICEDEG).

[57] Adipridhana M.G., Galinium M., Ipung H.P. Website Quality Assessment for Portal Hospital Indonesia Using Gap Analysis. Proceedings of the 2014 6th International Conference on Information Technology and Electrical Engineering (ICITEE).

[58] Vetter D., Ruhwinkel H., Raptis D.A., Bueter M. (2018). Quality Assessment of Information on Bariatric Surgery Websites. Obes. Surg..

[59] Report PAPH (2020). Statistical Journal of the Polish Association of Private Hospitals (PATH). Private Hospitals Sector in Poland. http://www.szpitale.org/wp-content/uploads/2020/09/OSSP_raport_2020_www.pdf.

[60] Główny Urząd Statystyczny (Statistics Poland) Powierzchnia i Ludność w Przekroju Terytorialnym w 2020 roku. https://stat.gov.pl/obszary-tematyczne/ludnosc/ludnosc/powierzchnia-i-ludnosc-w-przekroju-terytorialnym-w-2020-roku,7,17.html.

[61] Government Website Official and Reliable Information on COVID-19 in Poland. Lista Szpitali Zakaźnych w Polsce 2020 (pl). https://www.gov.pl/web/koronawirus/lista-szpitali.

[62] Główny Urząd Statystyczny (Statistics Poland) Kategoria K22 Ochrona zdrowia, Grupa.G261 Szpitale. https://bdl.stat.gov.pl/BDL/metadane/podgrupy/261.

[63] Mahatody T., Sagar M., Kolski C. (2010). State of the Art on the Cognitive Walkthrough Method, Its Variants and Evolutions. Int. J. Hum. Comput. Interact..

[64] Google PageSpeed Insights. https://developers.google.com/speed/pagespeed/insights/.

[65] Blink Audit Tool. https://audyt.blink.pl/.

[66] Backlink Checker. https://www.iwebtool.com/backlink_checker.

[67] WAVE Web Accessibility Evaluation Tool. https://wave.webaim.org/.

[68] Mobile-Friendly Test (Bulk Testing Tool). https://technicalseo.com/tools/mobile-friendly/.

[69] Wayback Machine Internet Archive: Digital Library of the World Wide Web. https://archive.org/.

[70] (2020). Word Cloud Generator. https://www.jasondavies.com/wordcloud.

[71] Dobbins M., Watson S., Read K., Graham K., Nooraie R.Y., Levinson A.J. (2018). A Tool That Assesses the Evidence, Transparency, and Usability of Online Health Information: Development and Reliability Assessment. Jmir. Aging.

[72] Cheng C., Dunn M. (2017). How Well Are Health Information Websites Displayed on Mobile Phones? Implications for the Readability of Health Information. Health Promot. J. Aust..

[73] Benito-Osorio D., Peris-Ortiz M., Armengot C.R., Colino A. (2013). Web 5.0: The Future of Emotional Competences in Higher Education. Glob. Bus. Perspect.

